# Supplementary material for: Rupestonic Acid Derivative YZH-106 Promotes Lysosomal Degradation of HBV L- and M-HBsAg via Direct Interaction with PreS2 Domain
Source: Viruses. 2024 Jul 17;16(7):1151. doi: 10.3390/v16071151 (PMC11281537; doi:10.3390/v16071151)
Supplement: Supplementary file 1 [file viruses-16-01151-s001.zip › Supplementary Information.pdf]

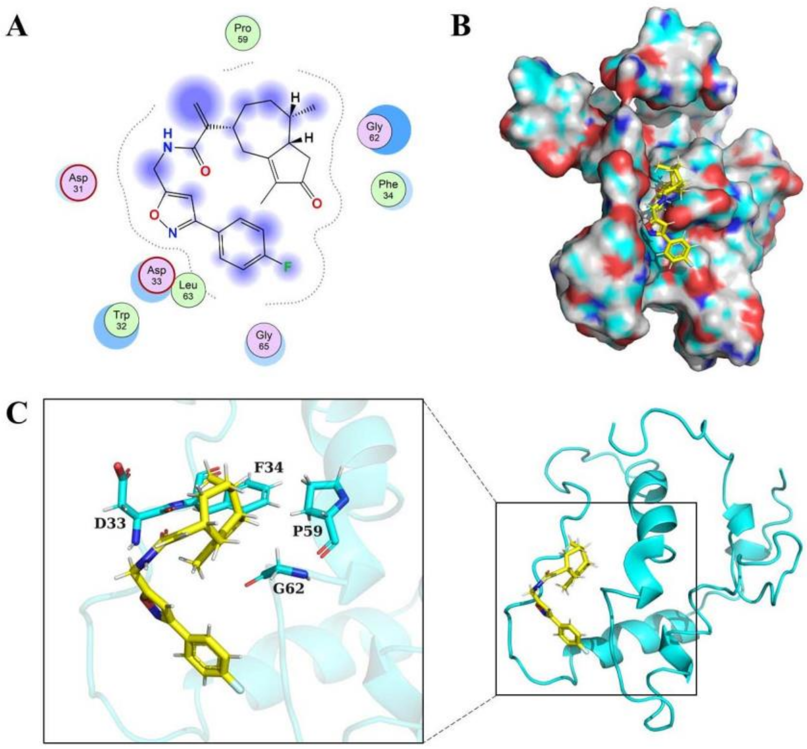

Figure S1. The binding mode of YZH-106 and PreS1. (**A**) The 2D binding mode of YZH-106 and PreS1. (**B**) The surface binding mode of YZH-106 and PreS1. (**C**) The 3D binding mode of YZH-106 and PreS1. The compound is colored in yellow. The surrounding residues in the binding pocket are colored in cyan. The surface of protein is colored in cyan, and the backbone of protein is shown in cyan cartoon.
